# Effect of *Rumex Abyssinicus* on preneoplastic lesions in dimethylhydrazine induced colon carcinogenesis in rats

**DOI:** 10.1186/s12906-015-0883-1

**Published:** 2015-10-15

**Authors:** Biniyam Girma, Getnet Yimer, Eyasu Makonnen

**Affiliations:** Pharmacology and Toxicology Course Team, School of Pharmacy, College of Health Sciences, Jimma University, Jimma, Ethiopia; Department of Pharmacology, School of Medicine, College of Health Sciences, Addis Ababa University, Addis Ababa, Ethiopia

**Keywords:** Chemoprevention, Colon cancer, Rumex abyssinicus, Aberrant crypt foci, Docking, Virtual screening

## Abstract

**Background:**

Cancer as a multistage process can be reversed or blocked by using chemopreventive agents. Colon cancer chemoprevention has been widely investigated using cyclooxygenase inhibitors and many other chemicals of synthetic or natural origin. This particular study was carried out to assess the colon cancer chemopreventive effect of hydro-methanol extract of *Rumex abyssinicus* rhizome on rats.

**Method:**

Colon cancer chemopreventive potential of hydro-methanol extract of *Rumex abyssinicus* rhizome was determined based on the number and multiplicity of aberrant crypt foci (ACF). Fifteen DMH (1, 2-dimethylhydrazine) treated and five untreated Wistar female rats were used. DMH was administered subcutaneously 30 mg/kg, after its pH was adjusted to 6.5–7. Treatment groups started receiving extract after six weeks of weekly DMH injections. The rats were divided in to four groups: Group 1 received only oral normal saline, Group 2 received DMH and normal saline, Group 3 and 4 received DMH plus 250 mg/kg and 500 mg/kg extract, respectively. Specific phytoconstituents of the plant, which were reviewed from original articles, were virtually evaluated for cyclooxygenase-2 (COX-2) inhibition. The binding energies and interactions of the phytochemicals from *Rumex abyssinicus* against COX-2 were determined by Autodock4.2.

**Results:**

There was a statistically significant reduction *(p-value < 0.05)* in the number of aberrant crypt (AC) and aberrant crypt foci (ACF) at both administered doses. However, significant association *(p-value > 0.05)* was not observed in reducing crypt multiplicity. The docking process resulted in estimated binding energies [−6.83 kcal/mol to −7.9 kcal/mol] which are closer to the positive controls or Non-Steroidal Anti-Inflammatory Drugs (NSAIDs) [−4.55 kcal/mol to −10.84 kcal/mol]. The phytochemical-COX-2 interaction indicated the involvement of key amino acid residues in inhibition of cyclooxygenase like ARG120, TYR355, TYR385, SER530 and GLY526.

**Conclusions:**

*Rumex abyssinicus* had demonstrated a chemopreventive potential at post-initiation stage. As the virtual screening data suggested, COX-2 inhibition by the anthraquinones in the extract could be one mechanism for the observed chemopreventive effect.

## Background

Colorectal cancer is one of the leading causes of cancer- related mortality globally. Therefore, it is a major public health challenge [1]. Curative and palliative therapies of colon cancer have commonly relied on surgery, chemotherapy and radiotherapy [2]. Apart from these major strategies, a relatively recent fourth method, which is called chemoprevention, is being pursued. It is the use of natural or synthetic compounds to block, reverse, or prevent the development of invasive cancers [3]. Non-Steroidal Anti-Inflammatory Drugs (NSAIDs) has been widely studied for chemoprevention of colon cancer. Despite their effectiveness, gastrointestinal perforation due to non-selective inhibition of cyclooxygenase enzymes has limited their use [4]. Even selective cyclooxygenase-2 (COX-2) inhibitors such as Celecoxib were removed from the market due to increased risk of heart attack and stroke [5]. Regardless of the challenges faced so far, COX-2 enzyme remains a prominent target for the development of chemopreventive agents. Natural products have also attracted great interest as a potential source of chemopreventive agents. Certain clinical trials and numerous *in vitro* studies showed phytoconstiuents like curcumin to have chemopreventive efficacy against colorectal cancer [2]. In eight weeks post-initiation animal study, red ginseng significantly reduced the incidence of ACF, which indicates potential for colon cancer chemoprevention [6].

*Rumex abyssinicus* Jacq (*polygonaceae*) is a large annual herb up to 4 m high. Its local Amharic name is ‘Mekmako’ [7]. In Ethiopia, it has been traditionally used for management of hypertension, inflammatory and painful conditions [8]. *Polygonacea* family and the genus *Rumex*, to which the study plant belongs, are rich in polyphenols like anthraquinones, flavanoids and terpens [9]. In particular, different studies found that *Rumex abyssinicus* to contain a number of anthraquinones [10–12]. In separate studies, some anthraquinones, which are also found in *Rumex abyssinicus,* showed COX-2 inhibitory activity [13, 14]. It has also been shown to have strong anti-inflammatory property, which indicates its potential as source of chemopreventive agents [15, 16]. In addition, extracts of *Rumex abyssinicus* and other *Rumex* species have shown antitumor activity against different cancer cell lines [17–19].

It is believed that inflammation is intimately linked to carcinogenesis. Over-expression of COX-2 is thought to be an early event in colon carcinogenesis and the development of other epithelial tumors. Thus, agents with anti-inflammatory properties and cyclooxygenase inhibition like NSAIDs are likely to exert chemopreventive action [6, 20]. Accordingly, we evaluated *Rumex abyssinicus* for colon cancer chemoprevention because of traditional claims and evidences of strong anti-inflammatory action plus evidences of potential cyclooxygenase inhibition.

In this study, we evaluated the colon cancer chemopreventive ability of crude hydro-methanol extract of *Rumex abyssinicus*. We used putative biomarkers of colon cancer named aberrant crypt foci to evaluate efficacy as a chemopreventive agent. In addition, we performed virtual screening of most abundant secondary metabolites of the plant like the anthraquinones against the commonly studied target COX-2. In doing so, we hoped to have insight to possible mechanism of action of the extract.

## Methods

### Experimental part

#### Animals

Wistar female rats weighing 200–300 g were used in this experiment. The rats were housed in a group of five per cage in a standard polyethylene cage. They were kept under ambient temperature and humidity. Day and night cycle was maintained at 12 h each. Food and drinking water were provided *ad libitum*. Body weights were monitored weekly throughout the study period. After acclimatization for a week, the rats were divided in to four groups, five in each group.

### Plant materials

#### Collection and extraction of plant material

The rhizomes of *Rumex abyssinicus* were collected from Addis Ababa. Then, a specimen was authenticated and deposited by National Herbarium, Biology Department, Addis Ababa University. The rhizomes were sliced to smaller pieces and dried at room temperature under shade. The pieces were then powdered and extracted. A kilo of powdered rhizome was divided in to four batches, and each batch was placed in a 5 L conical flask. Next, 80 % methanol was added to each flask up to a volume sufficient to fully cover the powder inside. It was left to macerate for 48 h with occasional shaking. The extract was then filtered and, the marc was re-macerated twice using the same solvent to exhaustively extract metabolites. The methanol and water, in the extract, were removed by rota vapour and lyophilizer, respectively. Finally, the dried extract was packed in a plastic bag and stored in a dry place at room temperature. The percentage yield of dried extract was found to be 16.4 % (w/w).

#### Dosing of extract

The dose selection was made based on a previous study on the diuretic and analgesic effect of *Rumex abyssinicus.* The dose used to produce analgesic effect was selected, as it may be linked with the anti-inflammatory activity of the extract. Accordingly, doses of 250 mg/kg and 500 mg/kg were considered. The rats were expected to tolerate the doses; because a preliminary study of 15 days found no acute toxicity and the lethal dose 50 (LD_50_) was greater than 5000 mg/kg [21]. Dose was calculated for individual rats based on their weekly weight which was taken at the beginning of each week. The dried extract was re-constituted in normal saline (0.9 % NaCl). A 100 ml stock solution with strength of 0.1 g/ml (w/v) was freshly prepared twice a week for the entire experiment.

#### ACF induction

DMH was prepared immediately before use, dissolved in 0.9 % NaCl containing 1.5 % EDTA as a vehicle at a final concentration of 10 mg/ml. The pH was adjusted to 6.5–7 with sodium hydroxide to ensure the stability of the chemical. The preparation was given subcutaneously, once a week, at a dose of 30 mg/kg body weight for six weeks to all except negative controls [22, 23].

#### Experimental protocol

The experiment was conducted for twelve weeks, after which all rats were killed by cervical decapitation. This method was intended to observe the effect of the extract on the progress of pre-neoplastic lesions. Thus, the extract administration was commenced six weeks after the first injection of DMH.

#### Treatment schedule

**Group 1:** Only 1.5 ml of Normal saline p.o everyday for six weeks.

**Group 2:** DMH (30 mg/kg body weight once a week s.c. for six weeks) + 1.5 ml of Normal saline p.o everyday for six weeks.

**Group 3:** DMH (30 mg/kg body weight once a week s.c. for six weeks) followed by Extract (250 mg/kg body weight in normal saline p.o starting six weeks after the first DMH injection till the end of 12th week) (POST- INITIATION— PI).

**Group 4:** DMH (30 mg/kg body weight once a week s.c. for six weeks) followed by Extract (500 mg/kg body weight in normal saline p.o starting six weeks after the first DMH injection till the end of 12th week) (POST- INITIATION— PI).

#### ACF scoring

The colons were evaluated for ACF according to Bird’s procedure. Each segments of the rats’ colons was fixed flat between filter papers in 10 % buffered formalin for at least for 24 h and then stained with methylene blue (0.2 % in saline) right before visualization. Staining was allowed to continue to 5–10mins. Finally, the aberrant morphology was observed at 40× magnifications using a light microscope with the mucosal side uppermost. ACF were distinguished from the surrounding crypts by their slit-like opening, elliptical shape, darker staining, increased size and pericryptal zone [24]. Chemopreventive response was assessed on the basis of AC, ACF, and ACF multiplicity.

#### Statistical analysis

Statistical analysis of data sets was done with SPSS version 16. Bivariate correlation was made using Pearson correlation coefficient. Pair wise comparisons were made using paired and independent t-tests. *p- Value* less than 0.05 were considered significant.

#### Limitation

Although virtual screening tools like autodock4.2 can reasonably predict binding energies and activities of ligands against different receptors, there are also a lot of limitations to their abilities. Hence, any claim of activity based on *In silco* studies needs to be backed by experimental findings, which is also the case in this particular study.

#### Ethical consideration

The handling of the animals was in accordance with the ethical standards of using animal subjects. It was also reviewed and approved by research ethics committee of Pharmacology Department, School of Medicine, Addis Ababa University.

### Docking part

The docking was done by using free software called Autodock4.2. The COX-2 [PDBe Code: 1CX2] enzyme 3D structure, which was used as a target receptor for the phytochemicals, was downloaded from http://www.ebi.ac.uk/pdbe/. Initially, we searched Google scholar and Pubmed for studies, which isolated and characterized specific phytochemicals of *Rumex abyssinicus* rhizome. After collecting the relevant literatures, we extracted the compound names reported to make a preliminary list. Then, the compounds in the list were reviewed for their solubility in 80 % methanol and fulfillment of all the criteria in Lipinski’s rule of five. Finally, a list of phytoconstituents was produced that we used for the *In silco* study. We downloaded the 3D structures of all the metabolites in the final list from PubChem. In addition, the 3D structures of all the ten positive controls, which were NSAIDs, were downloaded from PubChem. The pdb files of the ligands were converted in to pdbqt formats using ‘Quick ligand’ option in Autodock. The pdbqt format of the receptor was prepared by ‘Grid’ tab in the same tool.

The method was first validated by re-docking the SC58 to monomeric form of COX-2 both of which were obtained from the experimentally co-crystallized 1CX2 structure at 3 Å resolution. The docking was considered valid, only if the reference root mean square deviation (RMSD) was equal to or less than 2 Å [25]. Besides, the re-docked complex was checked for the presence of same ligand-receptor interactions like H-bonding described in the original literature. Further, the ability of the docking method to predict activity was validated by correlating the estimated binding energies from Autodock4.2 to experimental IC50 values of ten known COX-2 inhibitors (Positive Controls).

The receptor binding site to which the ligands were docked was defined by a grid box with X,Y,Z dimensions of 60,60,60 points, spacing of 0.375 Å and a grid center 23.295, 22.171, 16.173. The default docking parameters of Autodock4.2 were used except for the ga_run, which was reset to 50 runs. The Lamarkian Genetic algorithm was used as a search algorithm.

## Results

### Experimental part

This study investigated colon cancer chemopreventive potential of 80 % methanol extract of *Rumex abyssinicus* rhizome. We used 1, 2-dimethylhydrazine induced ACF as surrogate biomarkers of colon cancer. Our experiment assessed chemopreventive potential based on the number and multiplicity of abnormal crypts.

All the rats that received the carcinogen developed the pre-neoplastic lesions (*N* = 15). In the DMH only group (G-II), the mean ± standard deviation (SD) of ACs, ACF and crypt multiplicity was 103.60 ± 21.04, 24.80 ± 3.56 and 4.17 ± 0.64, respectively. Post- initiation group treated with 500 mg/kg extract (G-IV) showed the least number of AC, ACF and multiplicity and those treated with 250 g/kg (III) showed slightly greater number of abnormal crypts than 500 mg/kg groups. In comparison with the DMH only group, both the intervention groups significantly reduced the number of ACs and ACF *(p-value < 0.001 for 250 mg/kg vs. DMH only; p-value < 0.0001 for 500 mg/kg vs. DMH only)*. The extract reduced the incidence of ACs and ACF by more than fifty percent. However, none of the treatment doses significantly reduced crypt multiplicity *(p-value > 0.05 for 250 mg/kg or 500 mg/kg vs. DMH only)*. Similarly, comparison of the two doses didn’t show significant difference in terms of ACs as well as ACF reduction *(p-value > 0.05 for 500 mg/kg vs. 250 mg/kg)* (Table 1).

**Table 1:** Chemopreventive effect of hydro-methanol *Rumex abyssinicus* rhizome extract on the post-initiation of aberrant crypt foci (ACF) in six weeks DMH treatment induced colon carcinogenesis

Animal group	*N*	Treatment	AC	ACF	Crypt multiplicity
			Mean ± SD	Mean ± SD	Mean ± SD
Group I	5	Saline	0	0	0
Group II	5	DMH	103.60 ± 21.04	24.80 ± 3.56	4.17 ± 0.64
Group III	5	PI 250 mg/kg + DMH	41.40 ± 9.66^ac^	11 ± 3.00^ac^	3.83 ± 0.39^NSc^
Group IV	5	PI 500 mg/kg + DMH	33.20 ± 6.14^bc^	8.80 ± 1.64^ac^	3.78 ± 0.19^NSc^

*AC* aberrant crypt, *ACF* aberrant crypt foci, *crypt multiplicity* the mean total of AC counted/mean total of ACF, *DMH* 1,2-dimethylhydrazine, *N* number of rats, *PI* post-initiation, *SD* standard deviation

^a, b, NS^are all *P*-values where ^a^< 0.001, ^b^< 0.0001 and ^NS^ is not statistically significant

^c^Post-initiation 250 mg/kg OR post-initiation 500 mg/kg versus DMH

The mean weights of each group at the beginning and end of experiment were 222 g and 242 g for group 1; 232 g and 241 g for group 2; 234 g and 242 g for group 3; 233 g and 247 g for group 4, respectively. We did a paired *t*-test to identify if the extract given rats gained or lost significant weight. The result showed that neither of the treatment groups experienced significant weight change between the beginning and end of the study *(p-value = 0.140 for 250 mg/kg; p-value = 0.128 for 500 mg/kg)*.

### Docking part

The reviewing of literatures for specific phytochemicals of *Rumex abyssinicus,* which can be extracted with a hydro-methanol solvent, had resulted in nine compounds. All the metabolites were anthraquinones. They were Rhein, Chrysophanol, emodin, emodic acid, aloe-emodin, alizarin, physcion, damnacanthal and catenarin [10–12]. Lipinski’s rule of five, which can reasonably determine the oral bioavailability of drugs, was fulfilled by all the above mentioned compounds [26].

**Fig. 1:**
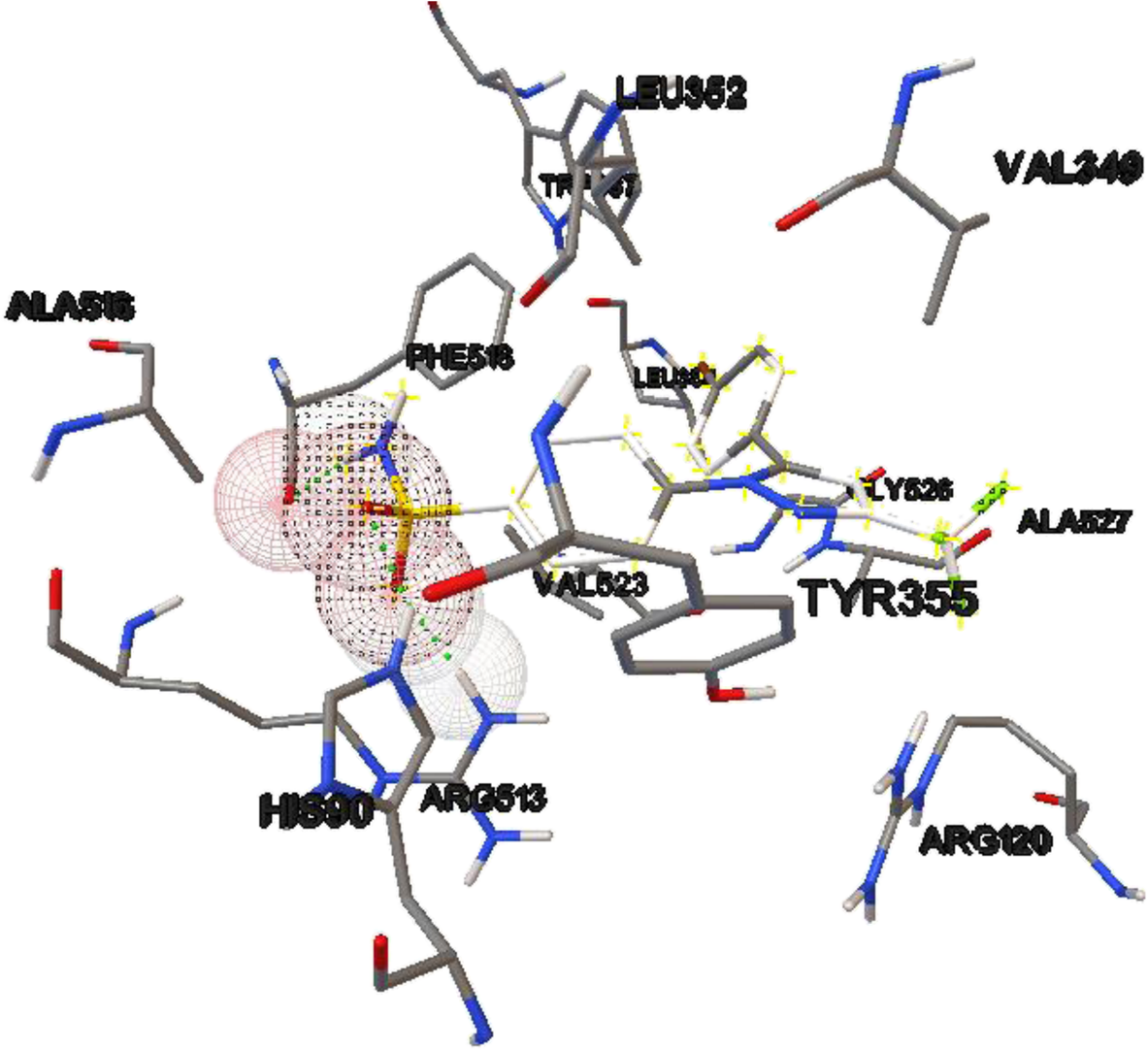
Hydrogen bond interaction between the sulfonamide group of SC58 and three amino acid residues, HIS90, ARG513 and PHE518, of Cyclooxygenase-2 (1CX2)

The re-docking of the SC58 to COX-2(1CX2) had resulted in a RMSD value of 1.5 Å, which indicated the success of the docking. The docking was also able to reproduce all the experimentally determined hydrogen bonds to the amino acid residues like HIS90, ARG513 and PHE518 (Fig. 1) [27]. Among the 50 runs only thirteen (26 %) were able to show the above mentioned ligand-receptor interaction. All the correct poses were found in the bottom quarter of reference RMSD values. The validation of the method with positive controls (Celecoxib, Aspirin, Meclofenamic acid, Ibuprofen, Flurbiprofen, Indomethacin, Meloxicam, Piroxicam, Nilusemide and Etodolac) had shown connection between estimated binding energy (ΔG) and activity. The –ΔG values from autodock4.2 and negative logarithm of inhibitory concentration 50 (IC50) from William Harvey Human Modified Whole Blood Assay (WHMA) had resulted in a statistically significant (*p-value = 0.003*) positive correlation (*r* = 0.835) [28].

The estimated binding energies of the positive controls including the original ligand, SC58, ranged from −4.55 kcal/mol to −10.84 kcal/mol. But, most produced binding energies from −7 to −10 kcal/mol. The binding energies of the phytochemicals were between −6.83 kcal/mol and −7.9 kcal/mol. Since the re-docking indicated that right ligand-receptor interactions were found in poses with relatively lower RMSD (bottom quarter), we had considered most repeated interactions of poses with RMSD in the lower 26 % as the correct ones. Consequently, the number of hydrogen bonds between the phytochemicals and COX-2(1CX2) ranged from zero to three. Except alizarin all compounds formed hydrogen bonding with the enzyme. Amino acids such as ARG120, TYR355, TYR385, SER530 and GLY526 were involved in hydrogen bonding (Table 2).

**Table 2:** Mean binding energies and interactions of docked phytochemicals of *Rumex abyssinicus* with Cyclooxygenase-2(1CX2)

Compounds	Mean (ΔG) (Kcal/mol)	Number of H-bond	Amino Acid Residues involved in H-bond
Rehin	−7.91	3	ARG120, TYR355, TYR385
Catenarin	−7.30	3	ARG120, TYR385, SER530
Emodin	−7.27	3	ARG120, TYR385, SER530
Emodic acid	−7.69	3	ARG120, TYR355, TYR385
Physcoin	−7.84	3	ARG120, TYR385, SER530
Chrysophanol	−7.48	1	GLY526
Damnacanthal	−7.78	2	TYR355
Alizarin	−6.83	nil	NONE
Aloe-emodin	−7.28	2	TYR355, GLY526

## Discussion

Carcinogenesis is a multistep process initiated from pre-neoplastic cells [29]. Experiments using pre-neoplastic lesions, which require less time to initiate carcinogenesis and use fewer study animals, provide an excellent end point to study chemopreventive agents [30]. In our study, we considered pre-neoplastic lesions named ACF.

The exact correlation of ACF and colon cancer is not quite clear. This has led to a number of investigations using ACF to correlate with colon cancer in different ways [31]. In the present study, we considered the number of individual AC, ACF and ACF crypt multiplicity to predict effect on colon cancer. The extract reduced AC and ACF significantly, which indicated it chemopreventive potential. Nevertheless, it was unable to significantly modify ACF multiplicity at any dose. It is widely believed that larger ACF are more likely to progress to invasive cancer than smaller ones [32]. So, the failure of the extract to truly influence the crypt multiplicity may hint an inferior chemopreventive ability.

Post initiation has more clinical relevance since it helps identify substances that prove useful in preventing the recurrence and progression of precursor lesions for colon cancer [33]. The significant reduction in AC and ACF numbers by the extract after the initiation of carcinogenesis could be attributed to its potent anti-inflammatory properties, which also interferes with prostaglandin synthesis [15, 16].

Inflammatory responses with COX-2 over expression are widely being related with different carcinogenesis steps of many cancers including colon [34]. Moreover, COX-2 mediates cell proliferation through production of free radicals. Generally, inhibition of these enzymes prevents cell proliferation, angiogenesis and induces apoptosis, and prevents formation of DNA adducts, which are all important in ending or altering carcinogenesis [4]. The estimated binding energy of the anthraquinones was very close and even sometimes greater than the positive controls used. Rhein, physcion, damnacanthal and emodic acid showed greater binding energy than known NSAIDs like aspirin (*ΔG = −4.55 kcal/mol)*, flurbiprofen (*ΔG = −7.35 kcal/mol)*, etodolac (*ΔG = −7.46 kcal/mol)* and meclofenamic acid (*ΔG = −7.53 kcal/mol)*. Given the very strong correlation between estimated binding energy and inhibitory activity (IC50) of the positive controls, proximity of ΔG values of the plant metabolites may indicate inhibitory potential. In addition, the ligands interaction to amino acids known to be involved in Cyclooxygenase inhibition by different NSAIDs such as ARG120, TYR355, TYR385 and SER530 will substantiate claim of COX-2 inhibition even more [35].

The above assertion was also observed in various experiments, though findings were variable. An *in vitro* assay which assessed COX-1 and COX-2 inhibition by natural quinones found that specific agents like damnacanthal were able to inhibit over 50 % of the COX-2 at higher concentration. However, apart from few other anthraquinones many of them were with little or no cyclooxygenase inhibitory activity [36]. Contrary to the previous study’s conclusion, a different evaluation had found COX-2 inhibitory activity by chrysophanol [13]. Another *in vitro* assay had also reported COX-2 inhibition by physcion and emodin obtained from *Rumex nepalensis*. In fact, emodin inhibited 78.1 % of COX-2 at 25 μM concentration, which was fairly close to celecoxib’s inhibition (88.6 %) at the same concentration [14]. All the above mentioned studies seem to agree on the fact that there is weak or no COX-1 inhibition by anthraquinones. This may be a huge advantage in promoting safe chemoprevention unlike many NSAIDs which can cause GI perforation due to COX-1 inhibition [4].

Finding a safe and effective chemopreventive agent is the main challenge currently. Since tolerability can be determined by observations such as clinical signs, reductions in body weight or a decrease in food consumption, perhaps, the lack of significant weight disparity, within the interventions, before and after extract administration, indicates tolerability of the doses and also may be a small but positive sign of safety combined with effectiveness used in the animals [37].

## Conclusions

In conclusion, hydro-methanol extract of *Rumex abyssinicus* rhizome demonstrated a chemopreventive potential, by effectively reducing the number of AC and ACF, at tolerable doses. The anthraquinones, which showed the potential to act as inhibitor of COX-2 and were among the dominant compounds in this polar extract, may alone or in combination have inhibited COX-2. But, further *in vitro* studies are required to conclude if each of the phytochemicals can truly inhibit the enzyme individually or in combination. In addition, other polyphenols like flavanoids, which are among the most commonly found metabolites in *Rumex abyssinicus*, may have contributed to the solid result either through the inhibition of COX-2 or other mechanism.
